# Non-Invasive Cardiac and Respiratory Activity Assessment From Various Human Body Locations Using Bioimpedance

**DOI:** 10.1109/OJEMB.2021.3085482

**Published:** 2021-06-01

**Authors:** Kaan Sel, Deen Osman, Roozbeh Jafari

**Affiliations:** Texas A&M University 14736 College Station TX 77843 USA

**Keywords:** Bioimpedance, hemodynamics, impedance cardiography, non-invasive, respiration

## Abstract

*Objective:* Bioimpedance sensing is a powerful technique that measures the tissue impedance and captures important physiological parameters including blood flow, lung movements, muscle contractions, body fluid shifts, and other cardiovascular parameters. This paper presents a comprehensive analysis of the modality at different arterial (ulnar, radial, tibial, and carotid arteries) and thoracic (side-rib cage and top thoracolumbar fascia) body regions and offers insights into the effectiveness of capturing various cardiac and respiratory activities. *Methods:* We assess the bioimpedance performance in estimating inter-beat (IBI) and inter -breath intervals (IBrI) on six-hours of data acquired in a pilot-study from five healthy participants at rest. *Results:* Overall, we achieve mean errors as low as 0.003 ± 0.002 and 0.67 ± 0.28 seconds for IBI and IBrI estimations, respectively. *Conclusions:* The results show that bioimpedance can be effectively used to monitor cardiac and respiratory activities both at limbs and upper body and demonstrate a strong potential to be adopted by wearables that aim to provide high-fidelity physiological sensing to address precision medicine needs.

## I. Introduction

Recent developments in integrated circuits and systems technology, mainly from the past decade, boosted wearable biomedical applications, and revealed unique seamless approaches that are sensitive to human physiological signals. These advancements revealed new opportunities to continuously and non-invasively monitor the operation of vital systems in the human body. The current state-of-the-art wearable systems provide certain cardiovascular health parameters such as heart rate (HR), heart rate variability (HRV), interbeat interval (IBI) with enhanced accuracy leveraged in smartwatches, smartrings, and electronic patches for health and fitness tracking purposes. However, for these wearable technologies to replace the invasive counterparts for medical diagnostic and prognostic purposes, precision medicine requires an accurate and high-fidelity assessment of more complex cardiac signs (*i.e.*, blood pressure - BP, cardiac output - CO) and respiratory parameters. This necessitates a thorough investigation of non-invasive modalities that are sensitive and specific to the physiological activities and can be integrated into a wearable form-factor to support seamless and continuous operations.

Bioimpedance sensing is a promising tool, leveraging deep penetration of very small electrical signals into the tissue to extract information that is modulated with the blood flow [1]–[3], respiration [4]–[7], hydration [8]–[10], muscle contraction [11], [12] and other physiological factors [13], [14]. The technique requires tissue stimulation with a very low-amplitude and high-frequency electrical current and capturing the established voltage signal with a low-noise sensing circuit. As each type of tissue in the human body (*e.g.*, fat, muscle, vessels) exhibit different electrical properties, the bioimpedance measurements are sensitive to the changes of the tissue composition in the sensing region [15]–[17]. Therefore, with proper placement of the bioimpedance sensors, it is possible to monitor the cardiac activity as well as the respiratory activities. The bioimpedance modality has formerly been investigated in its ability to assess cardiac parameters (*e.g.,* CO, [18]) and events (*e.g.,* pulse-wave velocity, [19], vessel volume change, [20]) with very large sensor sizes leading to impractical use. With the recent advances in sensor technology and miniaturization of technology nodes, the bioimpedance modality can be implemented in a wearable form factor. Although there are recent studies that measure bioimpedance mainly from the chest [2], [3], [8], upper arm [21] and wrist [22], [23], a comprehensive analysis of the bioimpedance signal from a larger set of human body locations (*i.e.*, foot, torso, neck, back) along with a detailed analysis of the signal morphology and characteristics is missing. In this manuscript, we share a complete analysis of the bioimpedance signal morphology from six different locations (*i.e.*, carotid, anterior tibial, ulnar, and radial arteries, side-rib cage, and thoracolumbar fascia) along with the investigation of the cardiac and respiratory information extracted from each location in comparison to the baseline finger-photoplethysmography (PPG) and belt-based respiration measurements.

This study provides important insights into opportunities associated with bioimpedance and their potential translation for clinical practice.

## II. Results

In presence of electrical stimulation, the body responds with an impedance dependent on the stimulation frequency (Supplementary I.A), a phenomenon called bioimpedance. Bioimpedance measurements at high frequencies (>1 kHz) resonate with the cardiac and other physiological activities of the human body. Based on the previous studies of the body response and injection frequency, our bioimpedance analysis uses an AC current of 10 kHz as the injected stimulation [3], [15]. The detailed discussion on the justification of the operation frequency is stated in Supplementary I.B. In the following part of this section, we will assess the relationship between the cardiac and respiratory activities, and bioimpedance modality at different body locations including anatomical explanations.

### A. Bioimpedance and the Arterial Cardiac Activity

Placing sensors on localized locations (*e.g.*, limbs) results in bioimpedance signals specific to the changes in the blood volume within the artery. The higher conductivity of blood cells, compared with fat, muscle, and bone tissues, creates the lowest impeding path for current flow. Therefore, the voltage signal acquired at the contact electrodes varies with temporal blood volume changes per cardiac cycle. Due to the quasi-periodic behavior of hemodynamic (i.e., blood-flow) activity with higher frequency (HF: >0.5 Hz & <6 Hz) compared with other physiological events (*e.g.,* respiration, body-fluid, and temperature shifts), the HF bioimpedance signal becomes quasi-periodic with the cardiac pulse, and the morphology carries the information of the blood volume, and its flow rate. At each cycle, highly conductive blood cells’ arrival at the arterial sensing site causes a drop in the magnitude of the HF bioimpedance signal. The amplitude and rate of change in the signal show the volumetric and flow velocity features of the pulse, which are affected by blood pressure, arterial compliance, and amount of blood that leaves the heart per pulse cycle (*i.e.,* cardiac output) [24]. We analyzed the morphologies of bioimpedance at different human body locations using a signal ensemble technique that depends on the simultaneously measured reference finger-PPG signals and explained in Supplementary I.C. In addition, we quantify the bioimpedance signal quality in reflecting the pulsatile activity in comparison to PPG.

#### 1) Ulnar and Radial Arteries

**Figure 1.**
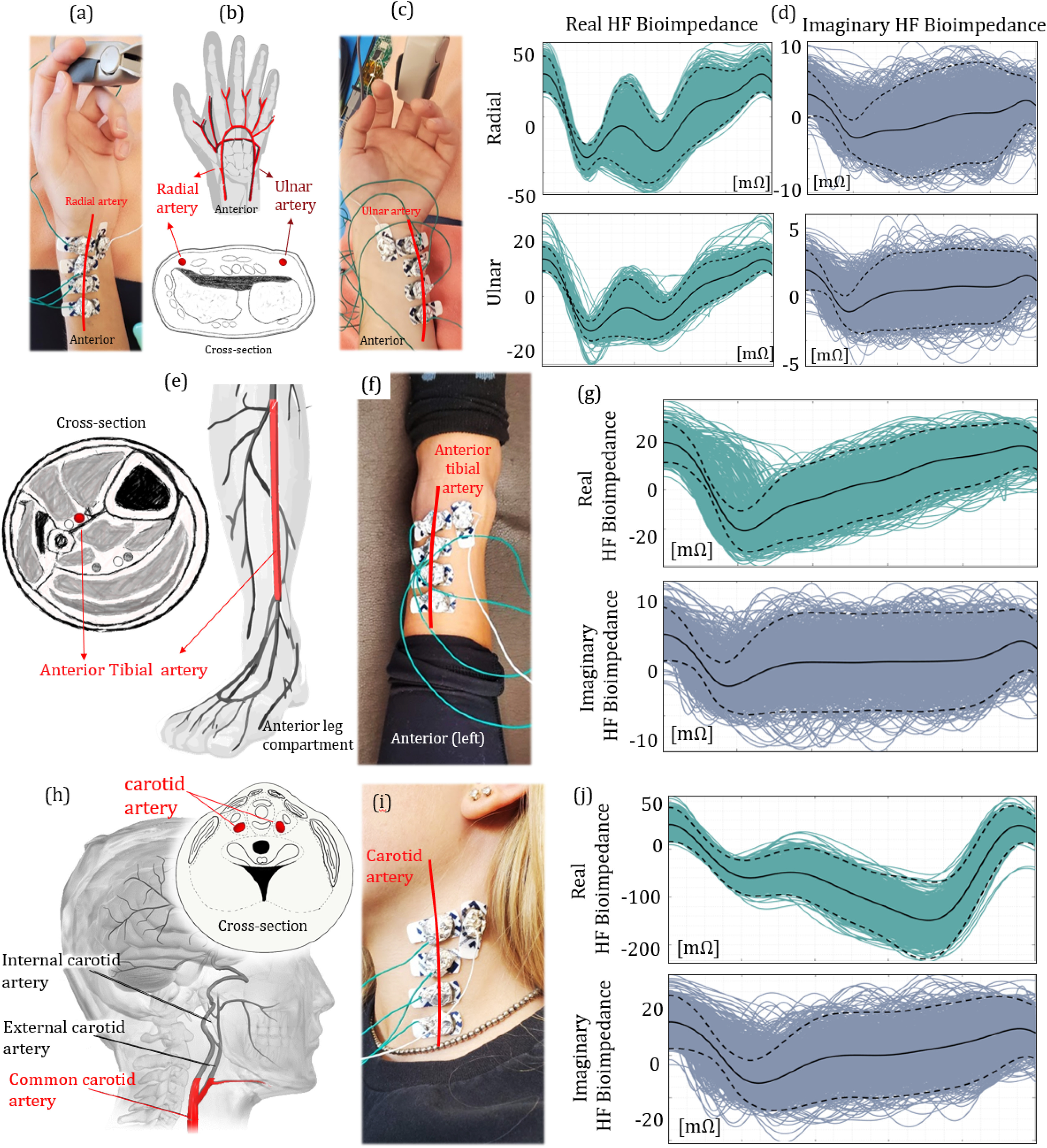
The bioimpedance signal analysis at different arteries. (a), (c) Electrode placement at the radial (left) and ulnar (right) arteries. (b) Cross-sectional view of the wrist, indicating the locations of both radial and ulnar arteries. (d) Real (left) and imaginary (right) ensemble HF bioimpedance signals obtained from the radial (top) and ulnar (bottom) arteries, showing the repeated quasiperiodic patterns. (e) The anterior leg anatomy, indicating the location of the anterior tibial artery. (f) Electrode placement at the anterior tibial artery. (g) Real (top) and imaginary (bottom) ensemble HF bioimpedance signals measured at the anterior tibial artery. The peak-to-peak amplitude of the HF bioimpedance signal is lower than that of radial artery under the same AC current injection due to hindered position of the tibial artery. (h) The cross-sectional and side view of the human neck with the carotid artery location. The red marked section is used to capture the bioimpedance signal. (i) Electrode placement at the common carotid artery. (j) Real (top) and imaginary (bottom) ensemble HF bioimpedance signals captured from the common carotid artery showing different morphology compared with other measured arteries, due to the late systolic carotid blood flow.

The wrist hosts two arteries (*i.e.*, ulnar and radial), that are derived from the proximal brachial artery. The wrist anatomy and the location of the arteries are shown in Fig. 1(b) and discussed in Supplementary I.D. Due to the proximity of both arteries to the skin, the wrist bioimpedance signal becomes sensitive to the pulsatile blood activity in the artery. We placed bioimpedance sensors at both locations proximal to the underlying blood flow as shown in Fig. 1(a) and 1(c), with 5 mm electrode separation. The real and imaginary morphologies of the ensemble HF bioimpedance signals are collected for over 900+ cycles, with cycles overlapped as shown in Fig. 1(d). A consistent double-trough pattern appears for the real bioimpedance signal. The first trough corresponds to the arrival of the blood at the sensing location during the systole [25]. The blood then travels through the hand with a reflection from the fingertip, forming the second trough in the signal [26]. Although the imaginary part shows a similar morphology on average, it shows a less consistent signal pattern due to a weak imaginary response to the blood flow at this operating frequency. The peak-to-peak HF bioimpedance signal amplitudes are measured as 100 mOhms and 20 mOhms for real and imaginary parts, respectively.

#### 2) Anterior Tibial Artery

The anterior tibial artery is located at the anterior compartment of the leg. The anatomical descriptions of the artery are shown in Supplementary I.D. The thicker skin and tissue along the base of the leg hinders the bioimpedance signal from arterial blood flow. In addition, the number of bones, ligaments, and tendons increases exponentially at the ankle joint. Therefore, we placed our electrodes on top of the ankle and sensed the blood flow through the bottom of the left anterior tibial artery, as shown in Fig. 1(f). The HF bioimpedance signal has 60 mOhms (real) and 15 mOhms (imaginary) peak-to-peak amplitudes with the same electrode separation, as shown in Fig. 1(g)-(j), and is also calculated for over 900+ cycles. The lower cardiac response at this location is as expected due to lower blood pressure at this arterial site compared with that in radial and ulnar arteries [27]. In addition, the lower magnitude of pressure causes a lower consistency (higher deviation from the mean) in the signal morphology. Nevertheless, pulse arrival is still indicated through the trough of the signal. The reflected wave is less visible compared to the wrist HF bioimpedance, due to the weaker signal magnitude and longer distance between the sensing location and the reflection at the phalanx.

#### 3) Left Common Carotid Artery

The common carotid arteries are located on both left and right sides of the neck. Specifically, we measure the blood flow through the left common carotid artery, which unlike the right common carotid artery branches directly from the aortic arch (Supplementary I.D) [28]. We place our electrodes at the base and midline of the neck where contact is optimal due to the restriction of head movement as shown in Fig. 1(i). The real HF bioimpedance response shows a different pattern with the second trough appearing at lower impedance than the first one due to the late systolic carotid flow, and this behavior is shown in Fig. 1(j) consistent for all 5 participants with the peak-to-peak amplitude averaging at 250 mOhms. This pattern matches with the previously reported blood inflow signal morphology captured from the carotid artery [29]. The imaginary response of the HF bioimpedance signal although higher with a 40 mOhms peak-to-peak average, shows a similar pattern with the other locations.

#### 4) Quality Assessment in Localizing Cardiac Activity

**Figure 2.**
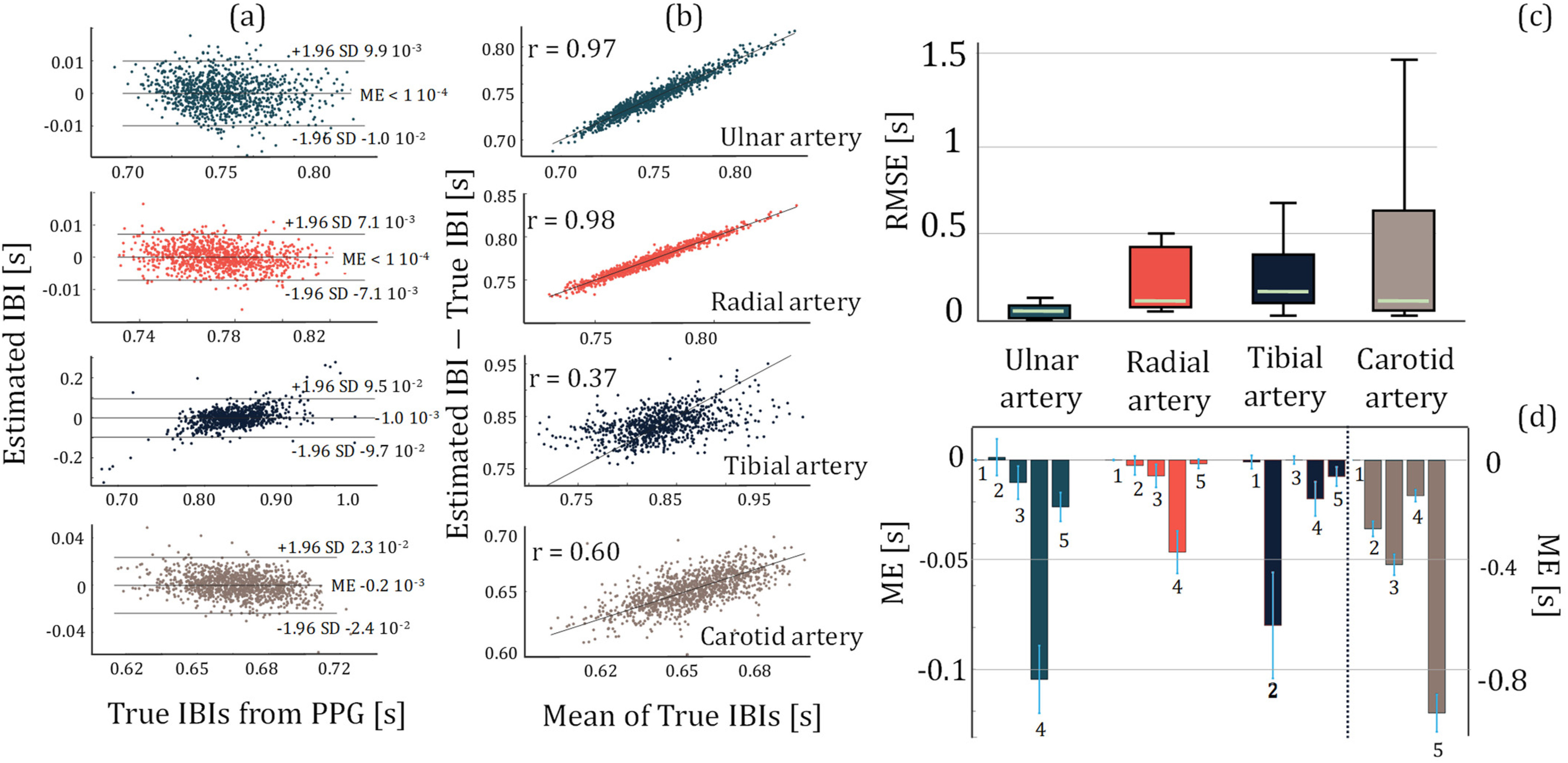
The statistical plots for the assessment of IBI estimation performance using HF bioimpedance. (a) Bland-Altman analysis plots for Subject 1, showing the mean error and 95% limits-of-agreement calculated from the estimated IBIs from the HF bioimpedance at four different arterial sensing sites and the true IBIs obtained from the finger PPG. (b) The Pearson's correlation analysis plots for Subject 1, showing the correlation between the estimated and true IBIs for each arterial sensing site. (c) The box plots showing RMSE distribution in IBI estimation from different arteries calculated over 5 subjects’ data. Ulnar artery shows the most consistent and highest performance. (d) The mean error distribution of each subject for different artery bioimpedance measurements. The numbers below the bars indicate the subject number. The left y-axis represents the ulnar, radial and tibial arteries, whereas the right y-axis represents the common carotid artery due to an order of magnitude higher error with the common carotid artery compared to the other arteries.

With the same electrode separation between different experiments, the amount of amplitude change in the HF bioimpedance signal provides a quantitative indication of the sensor sensitivity to the blood flow and blood volume changes from systole to diastole per cardiac cycle. Another indicator of the bioimpedance signal quality in reflecting cardiac activity is the performance in estimating the interbeat intervals (IBI) that are unique for each cardiac cycle. IBI represents the time interval between two successive heartbeats and can be calculated from the characteristic points in the HF bioimpedance signal (Supplementary I.H). Our IBI estimation performance is evaluated in reference to baseline finger PPG-derived IBI as shown in Fig. 2, where Fig. 2(a) and 2(b) show the corresponding Bland-Altman and Pearson's correlation analysis plots for a healthy participant measured at each arterial site, and Fig. 2(c) and 2(d) show the root-mean-square error (RMSE) and the mean error (ME) for all 5 participants calculated over 4 thousand cardiac cycles. We observe the highest IBI estimation performance at the ulnar arteries.

### B. Bioimpedance and the Respiratory Effort

The expansion and compression of the lungs within a period of breathing cause an immense variation in tissue composition and distribution, where the bioimpedance sensors placed in direct proximity to the chest can pick up this mechanical movement [4]. As the episodes of breathing occur at a slower rate compared with cardiac activity, it is possible to separate the contribution of the two activities of the bioimpedance signal based on their appearance at separate frequency bands. To capture the respiration effort we analyzed the low-frequency (LF: >0.045 Hz & <0.5 Hz) component of the bioimpedance for the sensing locations that are sensitive to the periodic motion of the lungs (*i.e.*, lower side-rib cage, top of thoracolumbar fascia).

When we place the bioimpedance sensors at locations where the movement from respiration activity is minimal, the signal becomes insensitive to the mechanical motion of the lungs. On the other hand, in the absence of the mechanical effect, another phenomenon named as cardiopulmonary coupling [30] becomes apparent. This phenomenon explains the influence of respiratory activity on the blood flow due to the impact of the intrathoracic pressure (also called intrapleural pressure) changes on the heart and therefore the hemodynamics, supervised by the autonomic nervous system (ANS) [31]. Details of this cardiac control mechanism are explained in Supplementary I.E. Placing the bioimpedance sensors alongside the arterial sites at the limbs results in a sensitive operation to the pulsatile activities in arteries rather than in surface capillaries. The influence of the respiratory activity on the blood flow is prominent and can be captured with the bioimpedance signal.

The rest of this subsection investigates the respiratory activity extracted from different body locations with bioimpedance due to both direct and indirect effects of respiration. At the section end, we share a quantification of the respiration signal estimation quality with respect to respiratory-belt measurements at three different breathing rates.

#### 1) Lower Side-Rib Cage and Top Thoracolumbar Fascia

**Figure 3.**
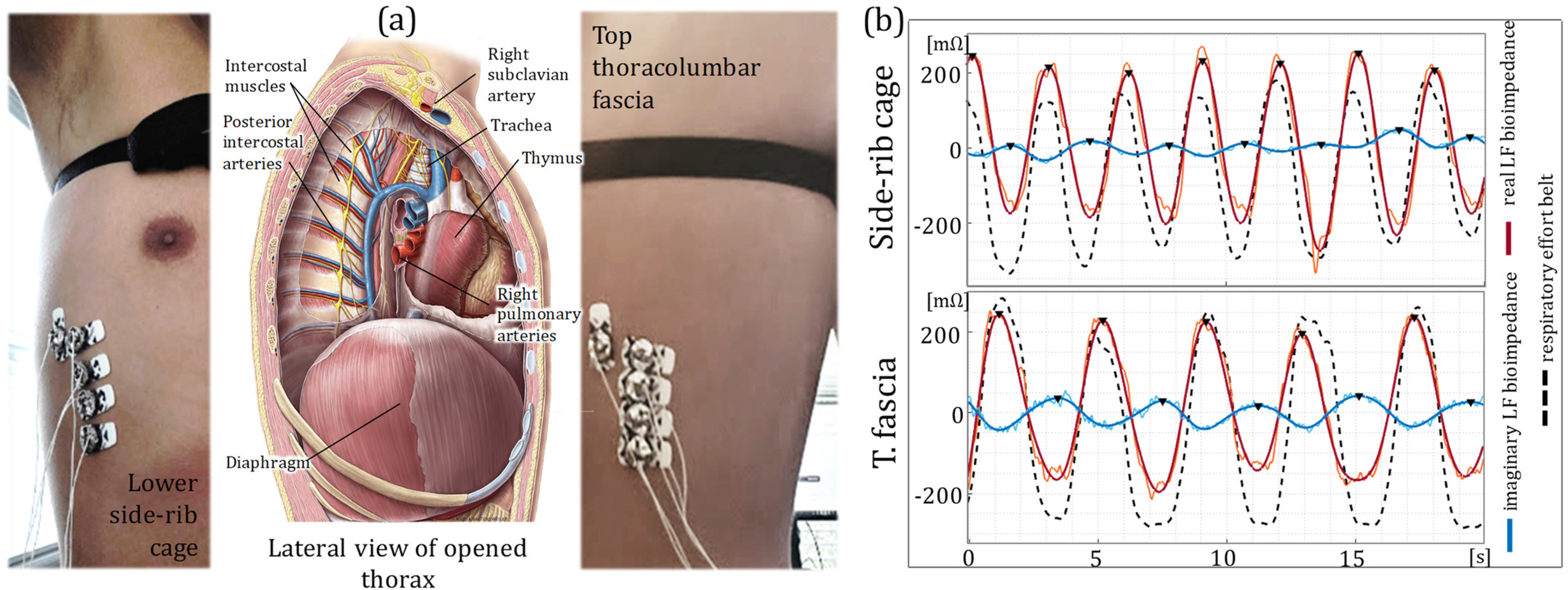
Thoracic bioimpedance measurements. (a) The anatomical description of the thorax and the thoracic cavity, showing the diaphragm, trachea, muscles and arteries. Electrode placement is shown for both lower side-rib cage (left) and top thoracolumbar fascia (right). (b) Time plot of the LF real (red) and imaginary (blue) bioimpedance signals and the reference respiratory effort belt (dashed black) for the side-rib cage (top) and top thoracolumbar fascia (bottom) locations. The bioimpedance signals show a strong agreement with the respiratory effort belt indicating a strong respiration influence over the thoracic bioimpedance measurements.

The thorax region spans from the base of the neck down to the diaphragm. Fig. 3(a) illustrates the lateral cross-section view of the thorax, with the anatomical descriptions shared in Supplementary I.D. Internally, the thorax houses the heart, lungs, and other cardiovascular/respiratory organs that contribute to changes in bioimpedance signals. Lung volume change, especially during respiration, is a significant factor in changing the direct bioimpedance along the thorax [4], [32]. Additionally, muscular movement is another significant factor that contributes to bioimpedance changes [4], [33]. The direct sensing of thoracic bioimpedance from the bottom of the thorax along the diaphragm yields a signal with significant influences from volume change and muscular movement controlled by the respiratory motion [34].

We placed our electrodes at two separate locations (lower side-rib cage and the top thoracolumbar fascia) to capture the thoracic bioimpedance signals as shown in Fig. 3(a). Fig. 3(b) shows the real and imaginary LF bioimpedance signals at each location in reference to the respiratory effort belt measurements, indicating the strong influence of the respiration to both sensing locations. On the other hand, neither of the locations show the HF variations related to the cardiac activity, due to their distal position to the blood activity.

#### 2) Indirect Respiration Estimation From Blood-Flow-Induced Bioimpedance

We investigate the indirect influence of the respiratory activity on the HF bioimpedance signals and capture high resolution blood flow at different arterial sites. We extracted three different modulations, namely the shift in baseline baseband (BB), change in amplitude (AM) and change in frequency (FM) from the HF bioimpedance (Materials and Methods, Supplementary I.I and II.A) and analyzed the respiratory band within each modulation type for all arterial sites (*i.e.*, ulnar, radial, tibial. carotid).

#### 3) Quality Assessment in Localizing Respiratory Activity

**Figure 4.**
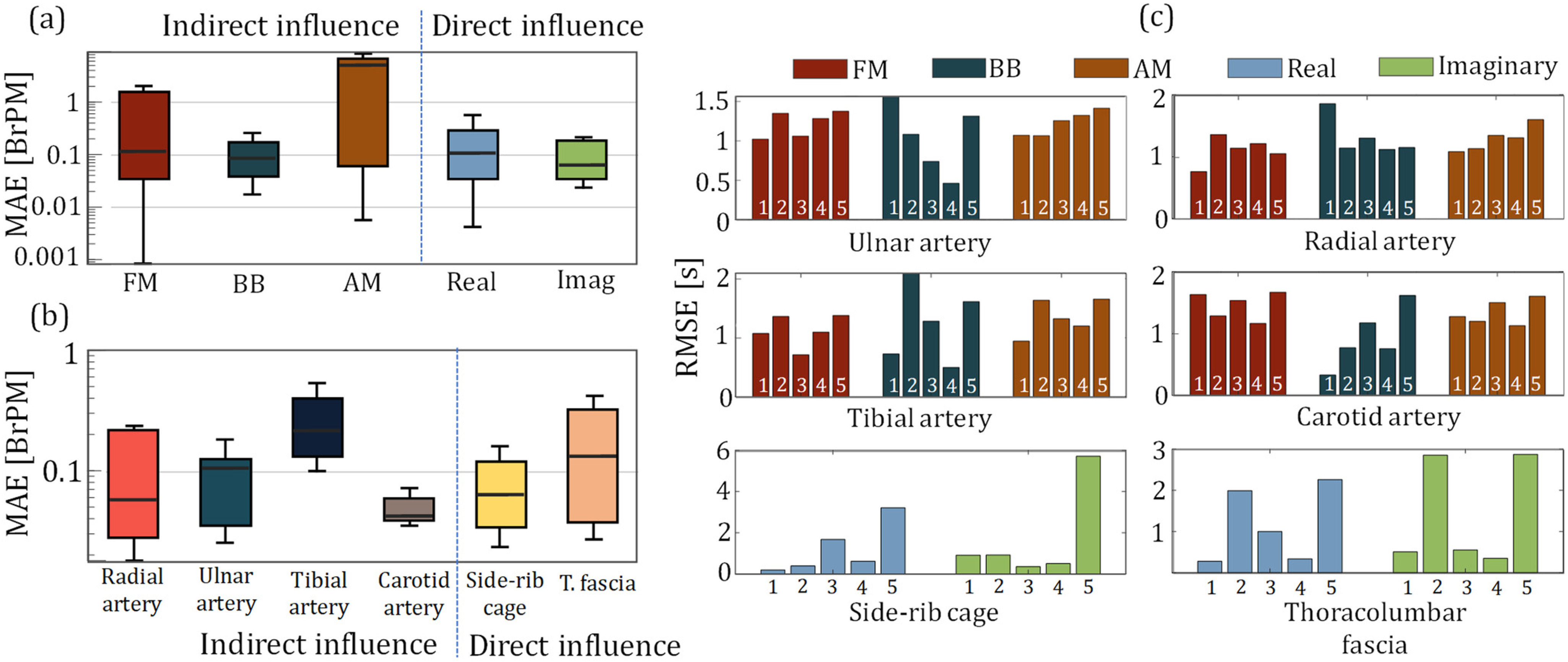
The statistical plots for the assessment of indirect and direct respiration influence quality over the bioimpedance, in reference to respiration effort belt. (a) mean absolute error (MAE) in estimating per trial breathing rate (BR) via the indirect modulation of the respiration over the arterial bioimpedance signals (FM - left, BB – middle, AM -right), along with the real (blue) and imaginary (green) components of the thoracic bioimpedance signals capturing the direct influence of the respiration. BB shows the highest performance and consistency in the BR estimation for the arterial bioimpedance signal, whereas the thoracic bioimpedance signals show higher average performance in BR estimation with their imaginary components (b) MAE for the BR estimation at different arterial and thoracic sensor locations. (c) RMSE in breath-by-breath inter-breath (IBrI) estimation for each modulation type for the arterial bioimpedance (top four) and for the real and imaginary components of the thoracic bioimpedance (bottom two) signals.

We extracted the peak points for both directly (*i.e.,* via thorax) and indirectly (*i.e.,* via arterial sites) obtained bioimpedance-based respiration signals, and calculated inter-breath intervals (IBrI) from the consecutive peak locations. Fig. 4(c) shows the RMSE in the estimation of the breath-by-breath IBrI at each sensing location with different respiratory modulations of blood flow, for all 5 participants. Fig. 4(a) and 4(b) show the mean absolute errors in estimating the average breathing rate for each modulation type per-trial and each sensing location separately. The results are from all the participant data, excluding the Subject 2 tibial arterial data and Subject 5 carotid arterial, and thoracic data due to poor electrode contact. For the latter analysis, only the BB for the arterial sites and imaginary component for the thoracic sites are used, due to their superior performance in representing the true respiration waveform. The results suggest that it is possible to capture the respiration rate on average with less than 1 breaths-per-minute (BrPM) error at each bioimpedance sensing sites.

## III. Discussion

Bioimpedance sensing is a non-invasive and powerful technique to assess human physiological signals due to its deep penetration into the tissues, leveraging its electrical nature. A thorough analysis of the bioimpedance sensitivity and specificity to physiological activities at different body locations is studied in this paper.

This study demonstrates an exploration of the bioimpedance signal over six different human body locations. We found that with proper placement of the bioimpedance sensors, the captured signal shows the patterns of the blood flow at the underlying major arteries that convey blood to the limbs. On the other hand, the signal shows different morphologies for different arterial sites, suggesting a location-specific flow that needs to be carefully assessed. In addition, the arterial activities captured at the radial and ulnar arterial sites demonstrated a better signal quality compared to that of the tibial and carotid arteries. The explanation for the decreased signal quality in the tibial artery can be associated with increased arterial depth (Supplementary I.D). There are two factors foreseen to cause a decrease in the bioimpedance signal quality obtained from the carotid artery: (i) the increased depth with the carotid artery compared to the radial and ulnar arteries and (ii) the muscular movement during respiration dominating the acquired impedance signals making blood flow harder to acquire from the neck (Supplementary I.D). Recent studies reveal the importance of morphological analysis to access more complex hemodynamic parameters (*e.g.,* arterial compliance) that are essential to understand one's health, yet currently require invasive interventions for monitoring. This study shows the repeated patterns in the bioimpedance signal at different locations over multiple participants. In the future, we plan to extend the analysis to reveal the reasons for morphological variances at different body locations and their correlation with the essential biometrics (*e.g.,* blood volume, velocity, pressure, vessel compliance), which are shown to be derived from the signal derived features [35], [36].

Our findings indicate a magnified respiratory response picked-up by the bioimpedance sensors at localized regions of the thorax, showing a potential for seamless respiration monitoring, unlike the conventional effort belts that introduce an inconvenient experience to the users. However, to avoid wrong estimations, other factors that affect the bioimpedance measurements should be well-identified. Due to the exploratory nature, this study does not provide a concrete method to extract the breathing rate (BR). Yet, the preliminary results in estimating the BR justify the potential of bioimpedance to replace inconvenient respiration monitors. The respiration rates examined in this study are typical rates for adults (*i.e.,* 12 to 16 BrPM). A validity study of the proposed modality at slower respiration rates will be useful to understand the limitations with bioimpedance based respiration monitoring.

This study includes young and healthy volunteers. Precision medicine and clinical practice require to develop a better understanding of the response of the bioimpedance signal, and from an extended number of participants along with a larger variety of demographics, which will be our future plan for this study.

## IV. Conclusion

This paper presents an exploration of the non-invasive bioimpedance modality across different human body regions. The results show bioimpedance can be effectively used to monitor cardiac and respiratory activities both at limbs and upper body. Therefore, the modality shows a strong potential to be adopted by various types of wearables (*e.g.,* smartwatches, smartbands, smartpatches) that aim to provide high-fidelity and high-resolution physiological sensing at multiple body locations to address precision medicine needs.

## V. Materials and Methods

### A. Experimental Procedure

5 presumably healthy participants (20 to 23 age range, 3 male, 2 female) volunteered in the experiments. Each subject gave their consent under the IRB approval IRB2017-0086D by Texas A&M University and acknowledged no significant respiratory or cardiovascular diseases. A total of 6 hours of bioimpedance data is acquired (Supplementary I.F).

### B. Bioimpedance Signal Acquisition and Post-Processing

A custom-developed printed-circuit board (PCB), Bio-Z XL, is used to measure the bioimpedance signal along with the changes due to physiological activity. The Bio-Z XL board operation is shared in Supplementary I.G. The obtained signals are then post-processed separately for arterial sensing locations and the thoracic cavity measurements, each explained separately in Supplementary I.H.

### C. Extraction of the Bioimpedance BB, AM, and FM Modulations

The bioimpedance modulation with the respiration is illustrated in Supplementary II.A. To obtain BB of the signal appearing at the 0.05 to 0.5 Hz frequency band, we applied a digital 6th order Butterworth band-pass filter. To acquire the AM and FM modulations, we used the HF bioimpedance signal envelope and the maximum slope points per cardiac cycle, respectively. The algorithms to extract each type of modulation are shared in Supplementary I.I.

### D. Statistical Analysis

We used mean error (ME), root-mean-square error (RMSE), and additional Bland-Altman (95% limits of agreement) and Pearson's correlation analysis (r) statistics to assess the IBI estimation performance from different arterial sites in reference to baseline IBI measurements.

For the assessment of the IBrI estimation performance from direct (*i.e.,* thoracic) and indirect (*i.e.,* arterial) measurements of respiratory activity, we calculated the RMSE statistics. For average breathing rate estimation performance analysis, we used the mean absolute error (MAE) metric.

## Supplementary Materials

This manuscript contains downloadable Supplementary Materials, to share a comprehensive elaboration of the bioimpedance modality, the related anatomical information necessary to build the connections between the measured signals and the physiology, the details of the bioimpedance acquisition system, and processing tools used in this study.

A comprehensive elaboration of the bioimpedance modality, the related anatomical information necessary to build the connections between the measured signals and the physiology, the details of the bioimpedance acquisition system, and processing tools used in this study.
