# Supplementary material for: Non-Invasive Cardiac and Respiratory Activity Assessment From Various Human Body Locations Using Bioimpedance
Source: IEEE Open J Eng Med Biol. 2021 Jun 1;2:210–7. doi: 10.1109/OJEMB.2021.3085482 (PMC8388562; doi:10.1109/OJEMB.2021.3085482)
Supplement: A comprehensive elaboration of the bioimpedance modality, the related anatomical information necessary to build the connections between the measured signals and the physiology, the details of the bioimpedance acquisition system, and processing tools used in this study. [file supp1-3085482.pdf]

Supplementary Materials

Non-Invasive Cardiac and Respiratory Activity Assessment from Various Human Body Locations Using Bioimpedance

Kaan Sel*, *Student Member, IEEE*, Deen Osman, and Roozbeh Jafari, *Senior Member, IEEE*

THE Supplementary Materials presented here are divided into two main sections. The first section includes the supplementary text to provide necessary details for the explanations shared in the main manuscript. The second section contains a supplementary figure that supports the analysis.

I. Supplementary Texts

A. Body response to electrical current at different frequencies

At direct current (DC) and very low frequencies (<100 Hz) epidermis shows an immense resistance to the flow of electrical signal (~MOhms), where only a negligible portion of this signal passes through this barrier [1]. Increasing the injection frequency exponentially lowers down the resistive barrier and the body's response becomes more capacitive as the signal starts to penetrate through the tissue membranes. This impending behavior is well-investigated in the literature and the modality is called bioimpedance [2]–[4]. It is shown previously that the bioimpedance of the tissues can be modeled with a combination of parallel and series resistance, R, and capacitance, C, where the former and the latter represent the inter-/intra-cellular fluids and the cell/tissue membranes, respectively. This allows the characterization of the frequency response of the body with the injected signal and choosing an optimum frequency range with sufficient penetration into the tissues, while causing no harm [5].

B. Effect of the injection frequency and amplitude considerations with the safety standards

Based on the previous studies of the body response and injection frequency, our bioimpedance analysis uses a signal injection at 10 kHz [6], [7]. There are three major considerations in choosing this operation frequency: (i) the lower-electrode skin impedance allowing the penetration through the skin with the electrical current, (ii) the allowance of an order of magnitude higher current injection at 10 kHz compared to <1 kHz according to the safety standards, (iii) the low contribution of flicker noise (1/f noise) at this frequency.

C. HF bioimpedance signal ensembling based on the quasi-periodic cardiac activity

We used the reference finger PPG waveform for the initial segmentation of the bioimpedance signal with the cardiac cycle. Next, we used the peak (PK), foot (FT) and maximum slope points (MPS) of the bioimpedance signal to finalize the ensembling. We used MSP to align the bioimpedance signals with the arrival of time, PK and FT to define the start and the end of the cardiac cycle and for time normalization of the ensembled signals for final grouping.

D. Anatomical view of the bioimpedance sensing locations

Wrist arteries

The wrist hosts two arteries, namely ulnar and radial that are derived from the brachial artery of the upper limb at the top for supplying the necessary circulation. From the two major blood sources within the wrist, the first oxygen-rich source, the radial artery, has on average a 2- to 3-mm diameter with its anterior wall being in 3 mm under the skin [8]. The artery gets deeper as it moves towards the hand and branches into a network of blood vessels. The other major blood source for the hand, the ulnar artery, travels along the small finger side of the hand, buried under the skin with 3 mm of depth [9]. Due to the close proximity of both arteries to the skin and the lower tissue impedance of blood due to the rich ionic solution a high percentage of the electrical current injected at the epidermis penetrates through the layers of skin and tissues, and reaches the arteries [10].

Anterior tibial artery

The anterior tibial artery spans from the end of the popliteal artery near the knee, travels down the leg anterior to the tibia, where it then ends near the ankle joint. The diameter of the artery spans from 2.5- to 5-mm [11]. The artery along with branching arteries supply the tibialis anterior muscle as well as the skin covering the tissue. We sense the blood flow through the bottom of the left anterior tibial artery, which then branches to become the dorsalis pedis artery supplying blood to the foot.

Left common carotid artery

The common carotid arteries are located on both left and right sides of the neck. The common carotid arteries are located on both left and right sides of the neck. The surrounding tissue such as the carotid sheath and sternocleidomastoid muscles protect the vital arteries which supply the majority of blood to the face and brain. The diameter of the arteries spans from 6- to 6.5-mm, with a length span along the sides of the neck [12]. Specifically, we measure the blood flow through the left common carotid artery, which unlike the right common carotid artery branches directly from the aortic arch. The anatomical placement of the artery is above the thoracic cavity and near the upper respiratory system [13]. Depending on electrode placement, the change in impedance can be due to arterial blood flow and respiratory movement during breathing. Henceforth, when collecting arterial blood flow from the left carotid artery the signal can be dominated by respiratory movement from surrounding organs.

Thorax and thoracic cavity

The thorax of the human body is composed of vital organs, which reside inside the thoracic cavity and are surrounded by protective muscle and bone tissue. The thorax region spans from the base of the neck down to the diaphragm. Along this region includes 12 ribs and thoracic vertebrae, muscle, and connective tissue, which all form the protective thoracic wall. Internally the thorax houses the heart, lungs, and other cardiovascular/respiratory organs that contribute to changes in bioimpedance signals when functioning. The movement of bodily gases, liquids, and solids expands the thoracic cavity changing the volume. Volume change, especially during respiration is a significant factor in changing the direct bioimpedance along the thorax. Additionally, muscular movement is the second most contributing factor to change in bioimpedance. The diaphragm located at the bottom of the thorax, is a large muscular organ responsible for the aid of volume expansion [14].

E. Respiration influence on blood-flow

During the process of respiration intrathoracic pressure will slightly fluctuate, with respect to volume change in the lungs and rest of the thorax. As a result of intrathoracic pressure change and the heart's anatomical location inside the cavity, cardiovascular mechanics (*e.g.* arterial pressure, cardiac output, and venous return) are influenced by the shift in pressure. Additionally, a physiological phenomenon occurs known as respiratory sinus arrhythmia, which is when an individual's heart rate increases during inhalation and vice versa. We can measure these changes in cardiac output and heart rate through arterial blood flow in various artery locations. The change in cardiac output can be observed through amplitude (AM) and baseline (BB) modulations where the volume of blood is altered due to change in intrathoracic pressure. Frequency modulation (FM) modulation of the heart rate due to respiratory sinus arrhythmia is observed as well, through arterial blood flow. Together these methods reconstruct respiratory signals observed in our Bio-Z signal.

F. Experimental analysis

5 presumably healthy participants (20 to 23 age range, 3 male, 2 female) volunteered in the experiments. Each subject gave their consent under the IRB approval IRB2017-0086D by Texas A&M University and acknowledged no significant respiratory or cardiovascular diseases. 10 mm-wide wet electrodes composed of foam and hydrogel were placed on top of the anterior tibial, common carotid, radial, and ulnar left side arteries with 5 mm separation. Additionally, sensors were placed on the lower lateral left side of the ribcage and medial left side of the upper lumbar region. Alternating current was injected into these locations with a frequency of 10 kHz and an amplitude of 0.8 mA. Three 4-minute trails of Bio-Z data collection was captured from each sensing location, where subjects were asked to breathe at 15, 20, and 25 breaths per minute respective to each trial. Subjects utilized an online metronome to breathe at the specified rate. Finger PPG with the TI-AFE4490SPO2EVM (Texas Instruments, USA) and respiration with the Vernier Respiration Belt (Vernier, USA) were simultaneously measured during bioimpedance data collection, used as standard baseline comparisons.

G. XL board multi-channel bioimpedance acquisition system operation

Bio-Z XL operation is based on an ARM Cortex M4 MCU. The MCU operates a 16-bit DAC converter (DAC8811, Texas Instruments, USA) to generate a sinusoidal voltage signal at an amplitude and frequency set by the user. The DAC output is connected to the negative terminal of a precision amplifier (OPA211, Texas Instruments, USA) with a capacitor to prevent DC signal injection, where the injection electrodes attached to the human body are connected to the negative feedback path of the amplifier. This ensures a stable and programmable current injection from the epidermis into the body.

The Bio-Z XL board sensing hardware uses low-noise discrete components to detect small variations (~50mΩ) in the bioimpedance signal. We leverage four-terminal sensing to pick up the voltage drop with the sensing electrodes only due to the stimulation of the underlying tissue impedance with the current injection, isolating the measurement from the effects introduced by the unideal electrode-skin interface. The signal is then amplified with a low-noise and low-power instrumentational amplifier (AD8421, Analog Devices, USA) with 20 dB and sampled with the high-speed and high-resolution 24-bit delta-sigma ADC (ADS1278, Texas Instruments, USA) with a sampling frequency of 93,750 Hz, after low-pass filtering with an anti-aliasing filter with cut-off frequency at 30 kHz. The overall system is tested with a calibration resistance and demonstrated less than 1mΩ root-mean-square (RMS) error, which is sufficiently lower than the target bioimpedance variations. The ADC samples are sent to the MCU through SPI, which then sends it to the PC through High-Speed USB Bridge for signal demodulation and post-processing.

To extract the bioimpedance from the raw ADC readings, we carry-out an I-Q demodulation, where we extract the in-phase and quadrature components of the signal to calculate the magnitude and phase of the bioimpedance. We apply a 2nd order Butterworth band-pass filter at [0.05, 6] Hz cut-off frequency to reject the out-of-band noise and the DC (stationary) component of the bioimpedance signal.

H. Bioimpedance signal post-processing per different body locations

Arterial

In the arterial sensing locations, the change in impedance is due to traveling blood flow with every heartbeat. The raw in-phase component of the Bio-Z signal from the arterial locations is then processed in two unique ways. The first applies a 6th order Butterworth high-pass filter at 0.05 Hz cut-off frequency to rid of DC offset all the while containing baseline shifts in the signal due to respiration. The second applies a 2nd order Butterworth band-pass filter at [0.5, 6] Hz cut-off frequencies, which rids of DC offset, out-of-band noise, and baseline shifts due to respiration. This signal is referred to as HF bioimpedance in the main manuscript.

Defining characteristics of each period in both signals are acquired, which includes peaks, foots, and maximum slope points (MSPs). The MSPs in the HF bioimpedance signals are used to acquire the baseline shifts due to physical movement and change in intrathoracic pressure during respiration . In

addition, the amplitude of the ΔBio-Z from peak to foot indicates blood volume change, which is the result of fluctuating intrathoracic and pulmonary arterial pressures . The foots are used to evaluate the inter-beat intervals (IBIs) that change in accordance with respiratory sinus arrhythmia [15]. The differences within these defining arterial factors are resampled at 4 Hz. To isolate the processed respiration signal from arterial blood flow, we apply an 6th order Butterworth band-pass filter with [0.05, 0.5] Hz cut-off frequencies to the resulting resampled signals.

Thoracic Cavity

The change in impedance along the thoracic cavity is due to fluctuations in lung volume, pressure, and movement of the diaphragm during respiration. The raw in-phase and quadrature components of the captured Bio-Z signals from the thoracic cavity are both analyzed. The same 6th order Butterworth band-pass filter at [0.05, 0.5] Hz cut-off frequencies is used to isolate the respiration signals from both components. This signal is referred to as LF bioimpedance in the main manuscript.

I. Algorithms to extract BB, AM, FM

The combination of algorithms used to detect and process the bioimpedance respiratory data are as follows. The maximum slope points in our high pass filtered bioimpedance signal fc = 0.045 are utilized to track baseline shifts in our signal, as a means of detecting baseline modulation due to respiration and respective cardiac mechanics. To detect frequency modulated (FM) respiratory due to respiratory sinus arrhythmia the maximum slope points in the HF bioimpedance signal are once again used to calculate change in interbeat intervals (IBIs). Envelope detection is used in the HF bioimpedance signal to demodulate amplitude modulated (AM) respiration through time. Each of these methods of respiratory demodulation are then resampled to 4Hz and passed through a band pass filter fc = [0.08 0.05] to acquire respiration through time. The following respiration signal's IBrIs are calculated via x-axis intercept from positive to negative amplitude. For comparison of respiratory rates, the dominant frequency in the FFT from 0.1 to 0.5 Hz of bioimpedance derived and belt respiration signals were found using peak detection. The frequency was then multiplied by a constant of 60 to acquire respiration rate per minute. The algorithms used to acquire respiration and perform statistical analysis were done in MATLAB and Python, with utilization of multiple libraries.

II. Supplementary Figures

A. Blood-flow modulations with the respiratory influence

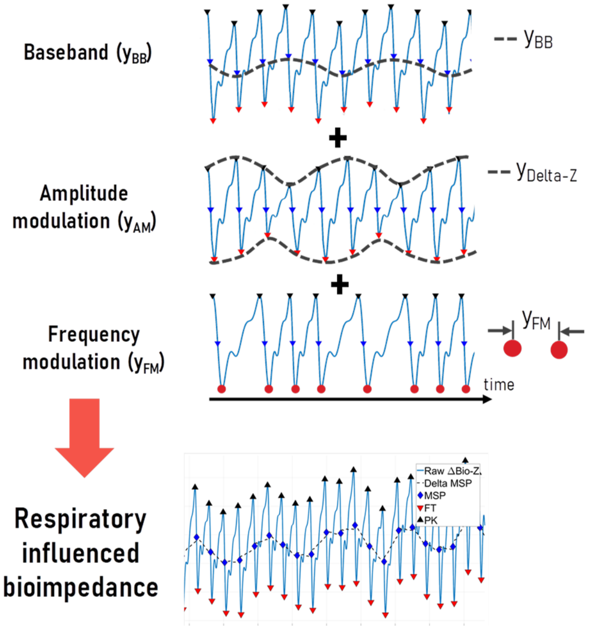

Supplementary Fig. A. Three main modulation of the bioimpedance signal with the indirect respiration influence.

References

[1] K. Sel, D. Kireev, A. Brown, B. Ibrahim, D. Akinwande, and R. Jafari, "Electrical Characterization of Graphene-based e-Tattoos for Bio-Impedance-based Physiological Sensing," 2019, pp. 1–4.

[2] T. Gerasimenko *et al.*, "Impedance Spectroscopy as a Tool for Monitoring Performance in 3D Models of Epithelial Tissues," *Front. Bioeng. Biotechnol.*, vol. 7, no. January, 2020.

[3] E. Alonso, R. Giannetti, C. Rodríguez-Morcillo, J. Matanza, and J. D. Muñoz-Frías, "A Novel Passive Method for the Assessment of Skin-Electrode Contact Impedance in Intraoperative Neurophysiological Monitoring Systems," *Sci. Rep.*, vol. 10, no. 1, pp. 1–11, 2020.

[4] T. Keller and A. Kuhn, "Electrodes for transcutaneous (surface) electrical stimulation," *J. Autom. Control*, vol. 18, no. 2, pp. 35–45, 2008.

[5] *Medical electrical equipment Part 1: General requirements for basic safety and essential performance ANSI/AAMI ES60601-1:2005/A1:2012.* .

[6] K. Sel, B. Ibrahim, and R. Jafari, "ImpediBands: Body Coupled Bio-Impedance Patches for Physiological Sensing Proof of Concept," *IEEE Trans. Biomed. Circuits Syst.*, vol. 14, no. 4, pp. 757–774, Aug. 2020.

[7] B. Ibrahim and R. Jafari, "Cuffless Blood Pressure Monitoring from an Array of Wrist Bio-impedance Sensors using Subject-Specific Regression Models: Proof of Concept," *IEEE Trans. Biomed. Circuits Syst.*, 2019.

[8] S. Beniwal, K. Bhargava, and S. K. Kausik, "Size of distal radial and distal ulnar arteries in adults of southern Rajasthan and their implications for percutaneous coronary interventions," *Indian Heart J.*, vol. 66, no. 5, pp. 506–509, Sep. 2014.

[9] J. U. J. Y. Kim, Y. J. Lee, J. Lee, and J. U. J. Y. Kim, "Differences in the properties of the radial artery between Cun, Guan, Chi, and nearby segments using ultrasonographic imaging: A pilot study on arterial depth, diameter, and blood flow," *Evidence-based Complement. Altern. Med.*, vol. 2015, 2015.

[10] U. G. Kyle *et al.*, "Bioelectrical impedance analysis - Part I: Review of principles and methods," *Clin. Nutr.*, vol. 23, no. 5, pp. 1226–1243, Oct. 2004.

[11] A. V. Telang, M. Lone, M. Natarajan, and A. Vishwanath Telang, "A study of the internal diameter of popliteal artery, anterior and posterior tibial arteries in cadavers," *Orig. Res. Artic. Indian J. Clin. Anat. Physiol.*, vol. 3, no. 3, pp. 287–290.

[12] J. Krejza *et al.*, "Carotid artery diameter in men and women and the relation to body and neck size," *Stroke*, vol. 37, no. 4, pp. 1103–1105, Apr. 2006.

[13] L. R. Caplan, "Basic Pathology, Anatomy, and Pathophysiology of Stroke," in *Caplan's Stroke*, Elsevier, 2009, pp. 22–63.

[14] A. Kudzinskas and A. L. Callahan, *Anatomy, Thorax*. StatPearls Publishing, 2020.

[15] K. Sel, A. Brown, H. Jang, H. M. Krumholz, N. Lu, and R. Jafari, "A Wrist-worn Respiration Monitoring Device using Bio-Impedance," in *Proceedings of the Annual International Conference of the IEEE Engineering in Medicine and Biology Society, EMBS*, 2020, vol. 2020-July, pp. 3989–3993.
